# Ba-Doped Iron Oxide as a New Material for NO_2_ Detection

**DOI:** 10.3390/ma6104801

**Published:** 2013-10-22

**Authors:** Christian Lopez, Chiara Baroni, Jean-Marc Tulliani

**Affiliations:** 1 Laboratory of Electrochemistry and Physical-chemistry of Materials and Interfaces, UMR 5279, CNRS-Grenoble INP-Université de Savoie-Université Joseph Fourier, BP75, 38402 Saint Martin d'Hères, France; E-Mail: christian.lopez@lepmi.grenoble-inp.fr; 2 Department of Applied Science and Technology, Politecnico di Torino, INSTM Reference Laboratory for Ceramics Engineering, Corso Duca degli Abruzzi 24, 10129 Torino, Italy; E-Mail: chiara.baroni@polito.it

**Keywords:** hematite, barium hexaferrite, NO_2_ detection, impedance measurements

## Abstract

Various compositions of barium-doped hematite between pure hematite (α-Fe_2_O_3_) and pure barium hexaferrite (BaFe_12_O_19_) were synthesized by solid state reaction. The XRD analyses confirmed the progressive evolution of the two crystalline phases. Tests as humidity sensors show that the electrical resistance of samples containing high proportions of hexaferrite phase is strongly influenced. Electrochemical impedance spectroscopy (EIS) analyses under air or argon revealed an intrinsic semiconducting behavior for hematite and samples doped with 3 and 4 wt % equivalent BaO. The samples containing higher proportions of barium exhibited an extrinsic semiconducting behavior characterized by a variation of the conductivity with the oxygen partial pressure. This study allowed us to define the percolation threshold of the barium hexaferrite crystalline phase in the hematite matrix. The value was estimated to hematite doped with 5 wt % BaO, *i.e.*, 36 wt % of barium hexaferrite phase. EIS analyses under various NO_2_ partial pressures confirmed the sensitivity of these materials. The linearity of the response was particularly evident for the 5, 10 and 14 wt % samples.

## 1. Introduction

Nitrogen oxides (NO and NO_2_: NO*_x_*), released from combustion facilities and automobiles, are a main cause of air pollution. They are responsible for acid rains, photochemical smog and are also potentially eutrophying agents, *i.e.*, can cause an oversupply of nutrient in soils and water bodies. Therefore, they are known to be harmful to the environment, to people, and also to historical monuments and buildings. The current directive 2008/50/EC of the European Union and the future Decision 2011/850/EU (from 1 January 2014) on ambient air quality has set at 40 μg/m^3^ the annual limit value, and at 200 μg/m^3^ the hourly limit value, not to be exceeded more than 18 times in a calendar year, for the protection of human health against the effects of gaseous NO_2_ [1,2]. Therefore, reliable, simple, effective and low-cost methods to monitor them have been highly demanded for atmospheric environmental measurements and controls. 

Many of the systems usually employed for the monitoring of air pollutants are based on traditional photometric techniques like chemiluminescence [1], even if, more recently, electroanalysis techniques (amperometric approach), have been proposed and tested [3]. The main drawbacks associated to these techniques are represented by the use of expensive bench scale laboratory equipment including calibrating facilities.

Semiconducting metal oxide (SMO) sensors are one of the most widely studied groups of chemiresistive gas sensors due to their unique advantages such as low cost, small size, measurement simplicity, durability, ease of fabrication, and low detection limits (<ppm levels) [3]. Moreover, most SMO based sensors tend to be long-lived and somewhat resistant to poisoning [3]. The SMO undergoes reduction or oxidation while reacting with the target gas and this process causes an exchange of electrons at a certain characteristic rate, thereby affecting the sensor’s resistance and yielding a certain signal [3].

Concerning the sensing materials for NO2 detection, tungsten oxide based materials have received a great attention in the last two decades. For example, pure and doped with various metal oxides WO3 sensors have been used as potential NO*x* sensors [3]. WO3-based sensors produced by screen-printing can be highly responsive to NO2 down to 1 ppm, when operated at 250 °C [4]. WO3 thick films fired at 700 °C in Ar/O2 flow have also proved to be operated at 100°C with excellent properties, such as fast response times, saturated stable sensitivity and rapid recovery characteristics to NO2 gas in air [5]. WO3-based nanocrystalline (3.0–9.0 nm size) thick films sensors with TiO2, can be used successfully for detecting and monitoring of NO2 in exhaust gases in parts per million level at 350 °C [6]. Zinc oxide thin and thick films have been also extensively studied for more than two decades. Several methods have been used to fabricate ZnO films and also their physical properties depend greatly on the method and condition of deposition [7]. Screen-printed ZnO, SnO2 and Sb2O3 thick-films sensors sintered at 800, 1000 and 1200 °C showed high sensitivity and excellent selectivity for ppm levels of NO2 gas [8]. Very recently, hybrid ZnO tetrapods + titanyl phthalocyanine exhibited a high sensor response ($\frac{\Delta R}{R_{air}}\approx 56$) under 100 ppb of NO_2_ at room temperature, but with slow response and recovery times (several tens of minutes) [9]. In contrast, monocrystalline SnO_2_ nanowires were sensitive in the range 18.9–1000 ppm of NO_2_ at 250 °C with fast response and recovery times of 7 s and 8 s, respectively [10]. Gas sensors based on indium oxide nanowires, In_2_O_3_ or In*_x_*O*_y_*N*_z_* films grown by the metal organic CVD technique also showed good selectivity to NO_2_ with little interference from other gases [11,12]. In addition, Indium Tin Oxide (ITO) thin films were found to exhibit high sensitivity toward NO_2_ and NO associated to a good selectivity with respect to CO and CH_4_ [12]. TeO_2_ thin films proved to be effective in NO_2_ detection in the range of 1–120 ppm too [13]. The results showed the best sensitivity to NO_2_ at room temperature, but with a response time of about 6 min for 1 ppm to about 1.2 min for 120 ppm NO_2_ concentration and longer recovery times.

Carbon nanotubes [14], YSZ [15], SnO_2_, Nb or In_2_O_3_ doped hematite [16,17] have been also proposed for NO_2_ detection. Alkaline or earthy-alkaline-doped hematite (α-Fe_2_O_3_) materials have been investigated recently in the literature, as NO*_x_* sensors [18] and 5 wt % BaO addition to hematite seemed to lead to a promising sensing material. Therefore, the aim of this work is to study in more detail barium-doped hematite as an electrochemical sensor for NO_2_ detection.

## 2. Experimental Section 

α-Fe_2_O_3_ powder (Aldrich > 99%, particle size distribution below 2 m) was mixed in ethanol with barium nitrate used as precursor of 3, 4, 5, 10 and 14 wt % equivalent of barium oxide respect to hematite (Fluka > 99%), until stoichiometrical composition of BaFe_12_O_19_, in a planetary mill for 1 h. After drying overnight, the mixtures were uniaxially pressed at 370 MPa and calcined at 900 °C for 1 h. These samples were then planetary milled for 6 h in ethanol with polyethylene glycol (PEG 4000, Sigma-Aldrich, Milan, Italy) to increase the densities of the different samples. After drying overnight, the powders were ready to use. The grain size of the produced powders was then determined by means of a laser granulometre (Fritsch analysette 22). The different mixtures of Ba-doped α-Fe_2_O_3_ were pressed ad 370 MPa again and sintered at 1300 °C for 1 h. 

Geometrical density evolution in function of percentage of added barium oxide was studied and the samples were characterized by X-ray diffraction (PW1710, Philips Eindhoven, The Netherland), in the 5°–70° 2 theta range, after calcination at 900 °C and sintering at 1300 °C. The pellets were also observed by means of a scanning electron microscope (SEM, S2300, Hitachi Tokyo, Japan). 

Interdigitated gold electrodes (ESL 520A) were screen-printed onto the surface of the pellets of hematite and doped hematite fired at 1300 °C and the sensors humidity response was studied in the range 0%–100% relative humidity (RH) because it is known in the literature that water molecules can interfere in gas detection, both with respect to adsorption of other species and to surface catalysis [19,20]. In a chamber, compressed air was separated into two fluxes: one was dehydrated over a chromatography alumina bed, while the second one was directed through two water bubblers, generating, respectively, a dry and a humid flow. Two precision microvalves allowed to recombine the two fluxes into one by means of a mixer and to adjust the RH content while keeping constant the testing conditions, in particular a flow rate of 0.05 L/s. The relative humidity was not increased in a continuous mode but was varied by steps every 3 min. The measurements were performed at room temperature. A commercial humidity and temperature probe was used as a reference for temperature and RH values (Delta Ohm HD2101.1), accuracy: ±0.1% in the 0%–100% RH range and −50–250 °C temperature range. Each tested sensor was alimented by an external alternating voltage (*V* = 3.6 V at the rate of 1 kHz) and then constituted a variable resistance of this electrical circuit. A multimeter (Keithley 2000) was used to measure the tension *V_DC_* at the output of the circuit. The sensor resistance was determined by substituting them, in the circuit, by known resistances and then plotting a calibrating curve *R* = *f* (*V_DC_*) [21,22].

The electrical behavior of the materials was studied by AC impedance spectroscopy (4192A LF Impedance Analyzer, Hewlett Packard, Palo Alto, CA, USA) after painting platinum electrodes (ESL 5545, Electro-Science Laboratories, King of Prussia, PA, USA) onto the two faces of the pellets (1 cm in diameter and 1 mm in thickness). The measurements were performed in a furnace under dry synthetic air (Messer air 80/20) and argon (Messer argon 5.0), between 100 and 700 °C, in the 5 Hz–13 MHz frequency range. Excitation voltage was fixed at 100 mV. The previous samples were also studied under NO_2_. AC impedance measurements were performed in a mixed flux of helium (Messer 4.6) and nitrogen dioxide (Messer 1000 ppm NO_2_ 1.8 in N_2_ 5.0) under a constant flow rate of 40 mL/min and a 0–500 ppm range NO_2_ concentration. Excitation voltage was fixed at 100 mV in the 5 Hz–13 MHz frequency range.

Sensor response (*SR*) was calculated from impedance measurements considering the resistance at 1000 Hz. *SR* is a function of NO_2_ concentration and is determined as follows [23] (Equation (1)):

(1) $$SR=\frac{R_{\left(Pgas\right)}-R_{\left(Pgas\to 0\right)}}{R_{\left(Pgas\to 0\right)}}$$

*R*_(*Pgas*)_ represents the value of resistance in presence of the gas studied (NO_2_ in the present case) and *R*_(*Pgas*→0)_ the resistance without this gas.

## 3. Results and Discussion

### 3.1. Microstructure

**Figure 1:**
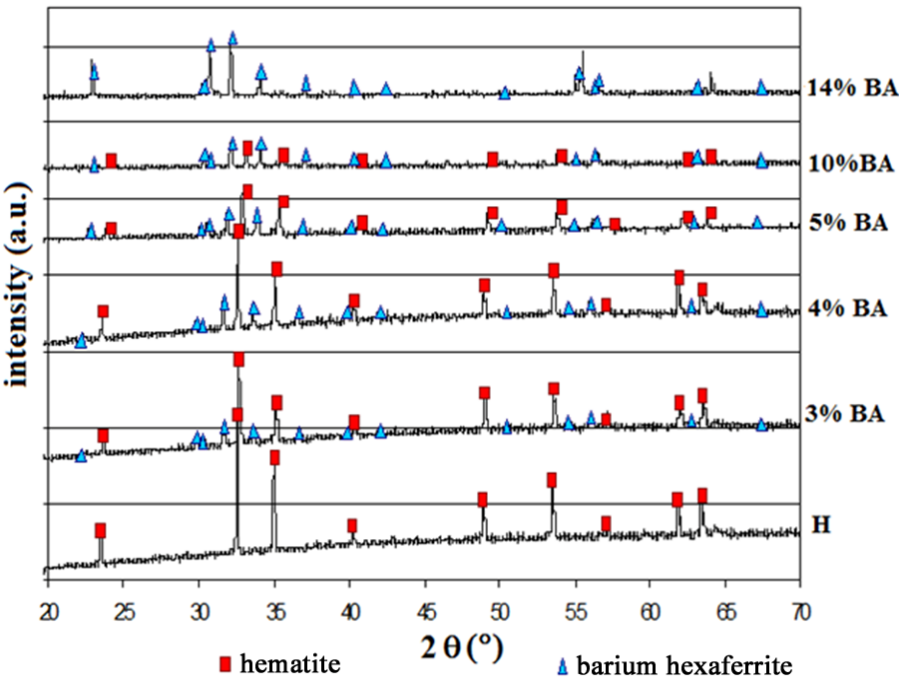
XRD spectra of various barium-doped hematite samples.

After the first thermal treatment at 900 °C and the 6 h planetary milling, the powders showed a mean diameter of 3.40 μm for 3, 4, 5 and 10 wt % equivalent of Ba-doped hematite samples, and of 9.00 μm for 14 wt % of Ba-doped hematite. These values are comparable to the mean diameter of the starting α-Fe_2_O_3_ powder (2 μm). X-ray diffraction patterns of the Ba-doped hematite after the first thermal treatment at 900 °C for 1 h and sintering at 1300 °C, confirmed the presence of two crystalline phases in all the samples: hematite (JCPDS card n.33-0664) and barium hexaferrite, BaFe_12_O_19_ (JCPDS card n.39-1433). As expected, we observe (Figure 1) the intensity of the barium hexaferrite peaks increasing with BaO additions from the pure hematite sample (H) to the pure barium hexaferrite sample (14% BA *i.e.*, α-Fe_2_O_3_ + 14 wt % BaO).

**Figure 2:**
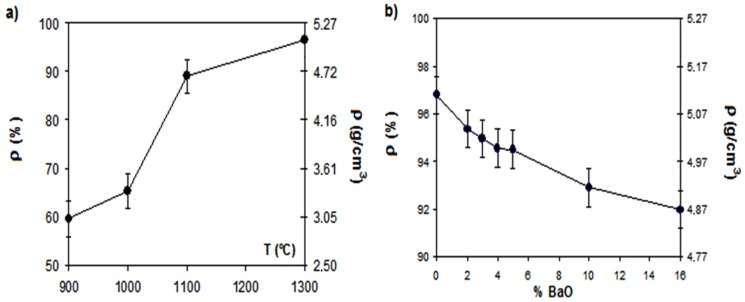
Geometrical density evolutions: (**a**) with temperature for 5% BA; (**b**) with barium oxide content for samples fired at 1300 °C.

Table 1 summarizes the six compositions studied in the present work. Theoretical densities were calculated considering that in all the mixtures the barium oxide was completely transformed into barium hexaferrite crystalline phase by reaction with hematite. The densities increase with increasing the sintering temperature (Figure 2a) but also decrease with barium oxide content (Figure 2b).

**Figure 3:**
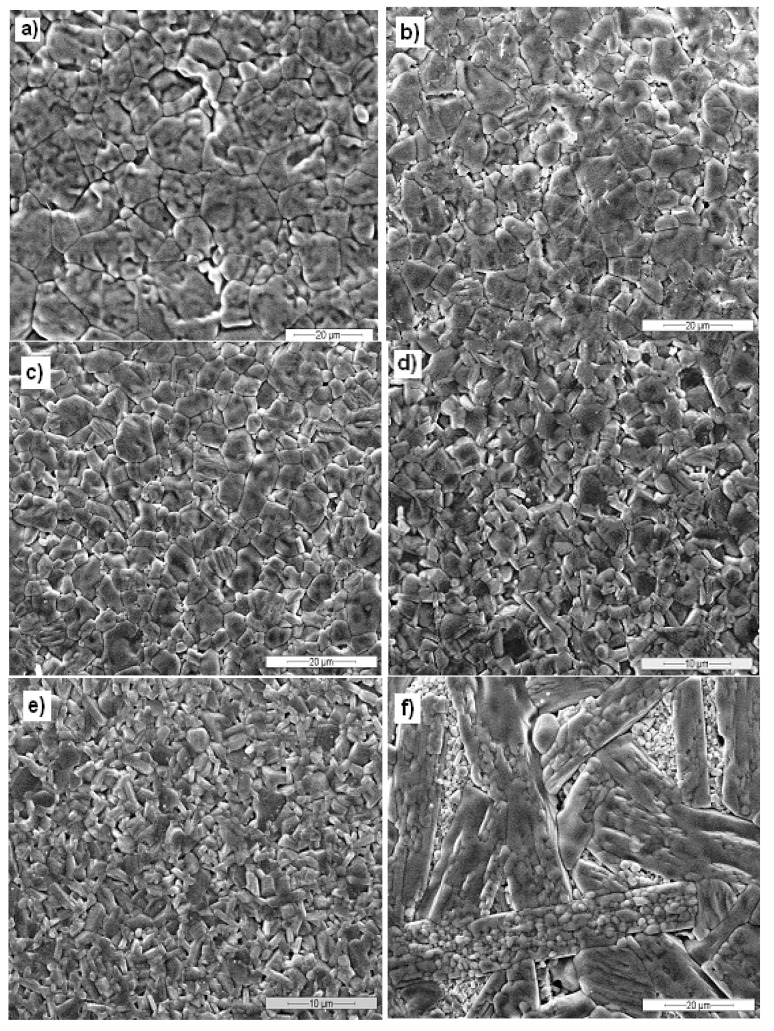
SEM micrographs of sintered samples: (**a**) H (1000×); (**b**) 3% BA (1000×); (**c**) 4% BA (1000×); (**d**) 5% BA (2000×); (**e**) 10% BA (2000×); (**f**) 14% BA (1000×).

Figure 3 illustrates the microstructures of pure hematite, 3% BA, 4% BA, 5% BA, 10% BA and 14% BA samples: the rather high densities of the materials fired at 1300 °C are confirmed. In the Ba-doped hematite samples, two different microstructures are present: a first one showing hexagonal grains, characteristic of hematite, and a second one characterized by lamellar grains, corresponding to the BaFe_12_O_19_ phase. 

**Table 1:** Density of the barium-doped hematite pellets with polyethylene glycol (PEG) addition.

Label	Sample	Expected BaFe_12_O_19_ (wt %)	Relative density (%)
H	α-Fe_2_O_3_	0	96.9
3% BA	α-Fe_2_O_3_ + 3 wt % BaO fired at 1300 °C	18.12	95.2
4% BA	α-Fe_2_O_3_ + 4 wt % BaO fired at 1300 °C	21.75	94.8
5% BA	α-Fe_2_O_3_ + 5 wt % BaO fired at 1300 °C	36.24	94.8
10% BA	α-Fe_2_O_3_ + 10 wt % BaO fired at 1300 °C	72.48	93.1
14% BA	α-Fe_2_O_3_ + 14 wt % BaO fired at 1300 °C	100.00	92.0

The grain sizes are rather different between the various samples (Figure 3): the grain size decrease when increasing barium hexaferrite content until 10% BA.

### 3.2. Humidity Sensitivity

The correlation between humidity sensor response and the material porosity is known in the literature, and it is possible to evaluate the pore radius at which capillary condensation occurs at different temperatures (*T*) by means of the Kelvin equation [19] (Equation (2)):

(2) $$r_{k}=\frac{2\gamma M}{\rho RT\ln\left(\frac{P}{P_{S}}\right)}$$

where *r_k_* is pore radius; *M* are respectively the water surface tension, density and molecular weight, while *P* and *Ps* are the water vapor pressures in the surrounding atmosphere and at saturation, respectively.

Because the porous structure of ceramics with open pores tends to favor water and gases adsorption and condensation, and though, on semiconducting materials these features are less critical [24], the efforts were oriented on the reduction of the sample porosity, as in general, dense ceramics show negligible humidity-sensitivity [25].

The retained solution was to increase the green density of the pellets by adding polyethylene glycol (PEG 4000) to the doped powder, during the 6 h planetary milling step and prior to uniaxial pressing. The samples were then sintered at 1300 °C and characterized like the previous ones. A significant increase of the density was then observed after PEG addition and sintering on the 5% BA composition (Table 2). 

Interdigitated gold electrodes were screen-printed on the surface of the different samples and the sensors humidity responses were studied in the range 0%–100% relative humidity (RH) at 20 °C. Humidity measurements were realized every three minutes.

**Figure 4:**
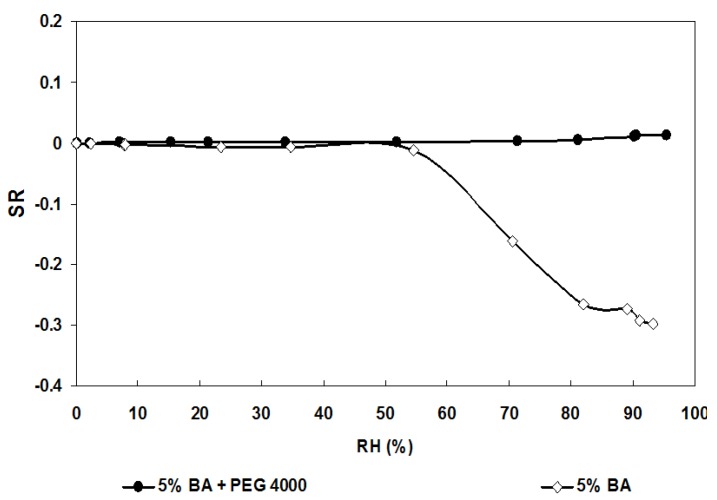
Humidity sensors (5% BA) response after 3 min of valve switch in function of RH, at 20 °C, for the samples fired at 1300 °C, with and without PEG 4000 addition.

As an illustration of the influence of density regarding the humidity sensitivity the electrical response of the 5% BA samples with and without PEG 4000 under water vapor (Figure 4) shows that the pellet with PEG (*R* = 1400 Ω) is quite insensitive to humidity. This result validates our choice to increase the density of the samples if we consider humidity as an interfering gas regarding nitrogen dioxide sensitivity.

**Table 2:** Density of the 5 wt % barium-doped hematite pellets with and without PEG addition.

Sample	Geometrical density (g/cm^3^)	Relative density (%)
5% BA	4.32 ± 0.05	83.0
5% BA + PEG 4000	5.08 ± 0.05	95.0

**Figure 5:**
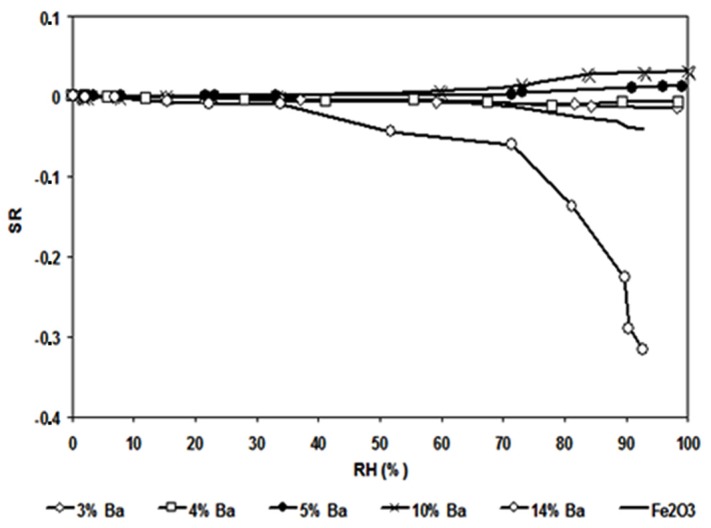
Humidity sensors response of various barium-doped hematite samples, after 3 min of valve switch, in function of relative humidity (RH), at 20 °C.

Subsequently, all the samples responses were characterized under humidity (Figure 5) at room temperature. They show no sensitivity to water vapor, with the exception of the 10% BA sample which exhibits a slightly increase of the SR above 70 RH % and the 14% BA sample for which an increase of the sensor response (negative values) is evidenced above 35 RH %.

### 3.3. Electrical Study

Impedance measurements were performed in dry synthetic air (20% O_2_) and under argon (<2 ppm O_2_), between 100 and 700 °C, in the 5 Hz–13 MHz frequency range.

**Figure 6:**
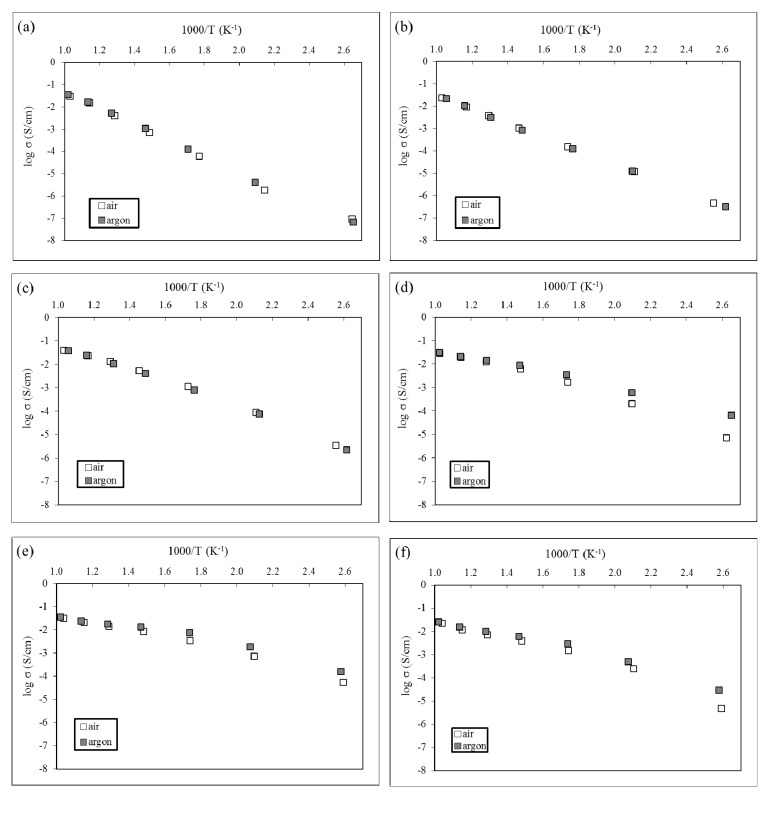
Arrhenius plots in air and argon of samples: (**a**) H; (**b**) 3% BA; (**c**) 4% BA; (**d**) 5% BA; (**e**) 10% BA; (**f**) 14% BA.

The conductivities evolutions in Arrhenius representations of pure hematite and the 5 compositions of barium-doped hematite samples showed that the samples behaved as semiconductors. Figure 6 shows two types of electrical behaviors. Pure hematite as well as 3% BA and 4% BA samples present a linear dependence of the conductivity with superimposition of the measurements under air and argon. 

The n-type semiconductor behavior of hematite is clearly reported in literature [26]. Oxygen vacancies ($V_{O}^{••}$) can be produced by heating this material following the disorder Equation (3) [27]:

(3) $$O_{O}^{X}=\frac{1}{2}O_{2(g)}+V_{O}^{••}+2e\prime$$

Gardner *et al.* [28] measured the oxygen deficit resulting from this equilibrium shift. Considering this structural disorder and the independence of the hematite conductivity regarding the oxygen partial pressure shown in the Figure 6a, we can conclude that this material is an intrinsic semiconductor [27,29]. The large value of activation energy measured in this work (Table 3) is the consequence of our experimental procedure where samples were sintered at 1300 °C and cooled under air laboratory. Under those conditions, the oxygen vacancies created during the heat treatment disappear by re-oxidation [28] and hematite becomes a slight n-type semiconductor [30]. The value of 0.71 eV obtained in the present work is close to the one reported by Gardner [28].

**Table 3:** Activation energies calculated from the linear regions of the Figure 6.

Sample	Activation Energy (eV)			
	Below 500 °C		Above 500 °C	
	Argon	Air	Argon	Air
H	0.71			
3% BA	0.61			
4% BA	0.54			
5% BA	0.36	0.51	0.27	
10% BA	0.35	0.40	0.26	
14% BA	0.43	0.52	0.34	

In the case of 5% BA, 10% BA and 14% BA samples, the Figure 6 exhibits two linear regions, below and above 500 °C (1000/T = 1.3 K^−1^): no influence of the oxygen partial pressure above 500 °C is observed, while a strong influence of the oxygen partial pressure below 500 °C is evidenced. The conductivities increase as the oxygen partial pressure decreases (the conductivities under argon are greater than the conductivities under air). This corresponds to the behavior of an n-type extrinsic semiconductor. 

Kim *et al.* [27] suggested a doping process with reduction of a part of Fe(III) to Fe(II), oxygen vacancies formation and concentration increase of negative charge carriers (electrons). These authors measured the electrical conductivity of pure and CdO-α-Fe_2_O_3_ system with various mol % of CdO in the 300–1300 °C temperature range and 10^−9^–10^−1^ atm oxygen partial pressure range. They also suggested that the semiconductivity became intrinsic for temperatures above 500 °C. We observe the same phenomenon and the activation energies deduced from the Figure 6d–f are close to the values obtained by Gardner *et al.* [28] on pure hematite in the same temperature range (0.1 to 0.3 eV).

The increase of barium percentages in hematite is correlated with the increase of the conductivity for the different materials. It is particularly evident since we compare the Figure 6a,f. The XRD analyses presented earlier underlined the presence of two crystalline phases: α-Fe_2_O_3_ (hematite) and BaFe_12_O_19_ (barium hexaferrite). If we consider that the Figure 6a represents the pure hematite electrical behavior and the Figure 6f the pure barium hexaferrite one, we can conclude that this latter phase is a better electronic conductor than the hematite phase. Considering now the intermediate compositions presented in the Figure 6b–e, it is noticeable that the transition of electrical behavior observed between the Figure 6c,d corresponds, respectively, to a composition transition between 21 to 36 wt % of the most conductive phase (barium hexaferrite) in the material. Therefore, for a composition below 36 wt % of barium hexaferrite, hematite is the main crystalline phase and the materials exhibit the intrinsic semiconducting behavior observed in the Figure 6a–c. The Figure 6c,d define a percolation threshold of the barium hexaferrite crystalline phase in the hematite matrix. Thus, the materials which composition exceeds 36 wt % of this latter phase, exhibit the n type extrinsic semiconducting behavior observed on the Figure 6d–f.

### 3.4. NO_2_ Sensitivity

**Figure 7:**
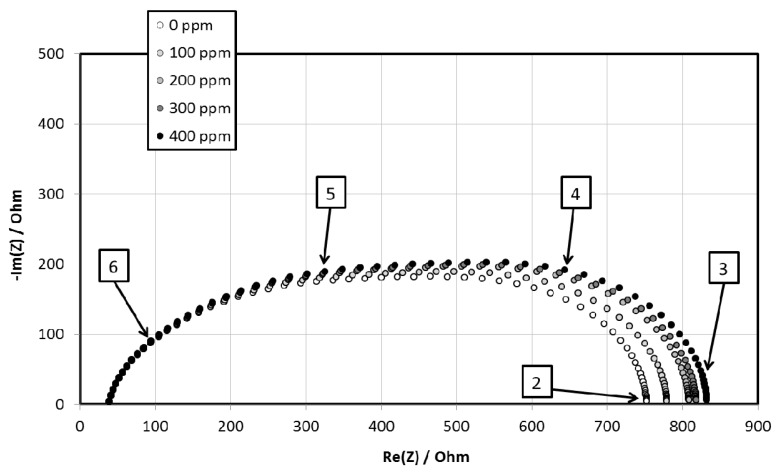
Impedance spectra evolution of 5% BA sample at 200 °C for various nitrogen oxide partial pressures.

The response to NO_2_ was investigated in a mixed flux of helium and N_2_/NO_2_ under a flow rate of 40 mL/min (0–500 ppm of NO_2_), between 60 and 350 °C, in the 5 Hz–13 MHz frequency range. As an example, Figure 7 represents the evolution of impedance spectra regarding the NO_2_ partial pressure. 

**Figure 8:**
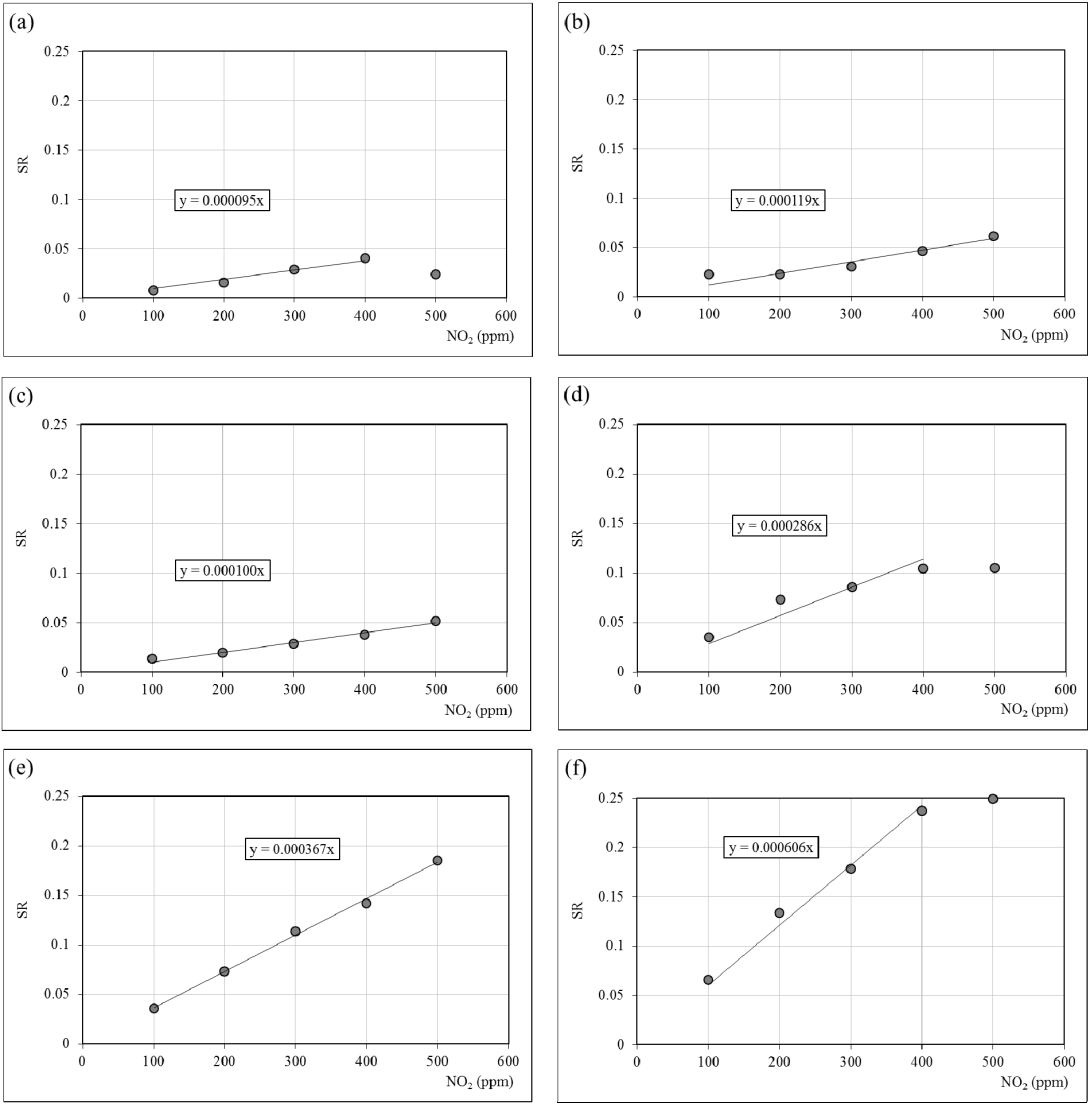
Sensor response (SR) evolution at 200 °C on the samples (**a**) H; (**b**) 3% BA; (**c**) 4% BA; (**d**) 5% BA; (**e**) 10% BA; (**f**) 14% BA in 0–500 ppm NO_2_. Resistance measured at 1000 Hz.

The gas composition clearly influences the low frequency impedance. Consequently the values of *SR* were calculated from resistance measured at 1000 Hz. Figure 8 represents the evolution of the *SR* (*P*_NO2_) of pure and doped hematite at 200 °C. We observed that for temperatures above 300 °C most of the materials exhibited a poor NO_2_ sensitivity while for temperatures below 200 °C the impedance of most of them were too high (and also the uncertainties) to permit a quantitative determination of the resistance. Therefore, considering the highest sensitivity *S* (slope of the representation *SR* (*P*_NO2_) gathered in the Table 4) and the lowest uncertainties, the optimal working temperature is estimated to 200 °C [16,31,32].

**Table 4:** Sensitivity (*S*) of hematite and various barium-doped hematite pellets measured at 200 °C.

Sample	*S* (ppm^−1^)	*R*(*P*_NO2→0_) (Ohm)
H	0.95 × 10^−4^	115,200
3% BA	1.19 × 10^−4^	11,485
4% BA	1.00 × 10^−4^	3943
5% BA	2.86 × 10^−4^	747
10% BA	3.67 × 10^−5^	695
14% BA	6.06 × 10^−4^	380

In the continuously regenerating diesel particulate filter (CRDPF) technology, NO_2_ is used to combust the soot collected in a particulate filter because it is a stronger oxidant than O_2_, promoting low temperature oxidation of soot in the range 200–500 °C [33]. This oxidation of carbon by NO_2_ is then achievable under normal driving conditions, particularly in heavy duty engine applications [34]. However, typically, NO_2_ is 5% to 15% of the total NO*_x_* in the diesel exhaust, but oxidation catalysts like Pt could oxidize NO to NO_2_ increasing NO_2_ concentrations to 50% of the total NO*_x_*, in the temperature range of 300–350 °C [33]. Then, emissions of NO*_x_* can be higher than 300 ppm [33,34], in function of the driving conditions, therefore, the materials studied in this work could be proposed as potential NO*_x_* sensors for diesel exhaust gases.

All the materials present a resistance increase with the NO_2_ partial pressure. This result has already been observed with hematite and various ferrites [16,32,35]. The 3% BA and 4% BA samples (Figure 8b,c) exhibit a SR evolution close to the pure hematite one (Figure 8a). Such a similarity was observed in the electrical study reported in the present paper. Otherwise, the Figure 8d–f show that beyond the percolation threshold described previously, the sensitivity to NO_2_ increases with the barium hexaferrite content in the sample. Nevertheless, considering the results of humidity sensitivity presented in the present paper the 5% BA sample seems to be the best compromise regarding the highest NO_2_ sensitivity with the lowest humidity interference.

In an n-type semiconductor, NO_2_ does not react with pre-adsorbed oxygen and resistance changes occur by a direct chemisorption process [36]. A series of reactions (Equations (4)–(8)) giving nitrates and nitrites is proposed in reference [37]:

NO_2_ + e^−^ (c.b.) ↔ NO_2_^−^ (4)

NO_2_ + V_O_^+^ ↔ NO_2_^−^ + V_O_^2+^ (5)

2NO_2_ + O_2_^−^ + e^−^ (c.b.) ↔ 2NO_3_^−^ (6)

2NO_2_ + O_2_^−^ + V_O_^+^ (c.b.) ↔ 2NO_3_^−^ + V_O_^+^ (7)

NO_2_ + O^−^ ↔ NO_3_^−^ (8)

In surface reactions (4) to (7), electrons from the conduction band (c.b.) are trapped when surface species are formed, but not in reaction (8). If nitrates are formed by the last mechanism, a further equilibrium has to be considered, that is to say NO^3−^ dissociation (Equations (9)–(10)):

NO_3_^−^ + e^−^ (c.b.) ↔ NO + 2O^−^ (9)

NO_3_^−^ + V_O_^+^ ↔ NO + 2O^−^ + V_O_^2+^ (10)

when O^−^ pressure increases, the resistance of the material also increases in an n-type semiconductor, as experimentally observed. 

## 4. Conclusions 

Ba-doped hematite were investigated as NO_2_ sensing materials. Electrical characterizations were performed by ac impedance spectroscopy under various temperatures and gas atmospheres. 

Pure hematite, 3 wt % and 4 wt % barium-doped hematite exhibit an intrinsic semiconducting behavior. They also show a poor NO_2_ sensitivity. 5, 10 and 14 wt % barium-doped hematite exhibit an n-type extrinsic semiconducting behavior. 

They are also good candidates for NO_2_ detection. The nitrogen dioxide sensitivity increases with the barium hexaferrite content in the material. Finally, the best compromise regarding the highest NO_2_ sensitivity with the lowest humidity interference is the 5 wt % barium-doped hematite.

The electrical study also underlines a percolation threshold of the barium hexaferrite crystalline phase in the hematite matrix. The value was estimated to 36 wt % barium hexaferrite. 
